# Ferumoxytol versus iron sucrose treatment: a post-hoc analysis of randomized controlled trials in patients with varying renal function and iron deficiency anemia

**DOI:** 10.1186/s12878-016-0060-x

**Published:** 2016-07-26

**Authors:** William E. Strauss, Naomi V. Dahl, Zhu Li, Gloria Lau, Lee F. Allen

**Affiliations:** AMAG Pharmaceuticals, Inc., 1100 Winter Street, Waltham, MA 02451 USA

**Keywords:** Ferumoxytol, Hemoglobin, Iron deficiency anemia, Iron sucrose, Chronic kidney disease

## Abstract

**Background:**

Iron deficiency anemia is highly prevalent in patients with chronic kidney disease and is often treated with intravenous iron. There are few trials directly comparing the safety and efficacy of different intravenous iron products.

**Methods:**

This post-hoc analysis pooled data from 767 patients enrolled in two randomized, controlled, open-label trials of similar design comparing the treatment of iron deficiency anemia with ferumoxytol and iron sucrose across patients with all stages of renal function. One trial was conducted in adults with CKD either on or not on dialysis and the second in adults with IDA of any underlying cause and a history of unsatisfactory oral iron therapy or in whom oral iron could not be used who had normal to no worse than moderately impaired renal function. Patients were categorized by chronic kidney disease stage (i.e., estimated glomerular filtration rate), and the primary efficacy endpoint was the mean change in hemoglobin from Baseline to Week 5.

**Results:**

The overall incidence of adverse events was numerically lower in ferumoxytol-treated patients compared to those treated with iron sucrose (42.4 vs. 50.2 %, respectively); the incidence of treatment-related adverse events was generally similar between the two treatment groups (13.6 vs. 16.0 %, respectively). Adverse events of Special Interest (i.e., hypotension, hypersensitivity) occurred at lower rates in those treated with ferumoxytol compared to those treated with iron sucrose (2.5 vs. 5.3 %, respectively). Overall, mean hemoglobin increased in both treatment groups, regardless of degree of renal insufficiency, although greater increases were seen among those with less severe kidney damage. Mean increases in hemoglobin from Baseline to Week 5 were significantly greater with ferumoxytol than with iron sucrose treatment in the subgroup with an estimated glomerular filtration rate ≥90 mL/min (Least Squares mean difference = 0.53 g/dL; *p* < 0.001). There were no other consistent, significant differences in hemoglobin levels between treatment groups for the other chronic kidney disease categories except for isolated instances favoring ferumoxytol.

**Conclusions:**

The efficacy and safety of ferumoxytol is at least comparable to iron sucrose in patients with varying degrees of renal function.

**Trial registration:**

(**CKD-201**; ClinicalTrials.gov identifier: NCT01052779; registered 15 January, 2010), (**IDA-302**; ClinicalTrials.gov identifier: NCT01114204; registered 29 April, 2010).

**Electronic supplementary material:**

The online version of this article (doi:10.1186/s12878-016-0060-x) contains supplementary material, which is available to authorized users.

## Background

Iron deficiency anemia (IDA) is the leading cause of anemia worldwide [1]. In the United States, IDA affects approximately 1 to 2 % of men and 2 to 5 % of women [2]. IDA is particularly common in patients with chronic kidney disease (CKD) [3–5].

Correction of the underlying cause of IDA and repletion of depleted iron stores are fundamental approaches to the treatment and management of IDA [6]. Intravenous (IV) iron plays a major role in the treatment of IDA across all degrees of renal function, and in particular for those with CKD, including dialysis-dependent CKD patients, and non-CKD patients unable to tolerate oral iron or in whom oral iron is either ineffective or contraindicated. According to the 2012 Kidney Disease Improving Global Outcomes clinical practice guidelines, a trial of IV iron is recommended in all adult CKD dialysis patients with anemia not on iron or erythropoiesis-stimulating agent therapy and in non-dialysis patients with CKD after a failed trial of oral iron [7].

The efficacy of IV iron supplementation in the treatment of IDA has been studied in patients with a variety of underlying conditions, including CKD, abnormal uterine bleeding, pregnancy, postpartum anemia, cancer, and gastrointestinal (GI) disorders, including inflammatory bowel disease and GI blood loss. However, few randomized head-to-head studies specifically comparing the relative safety and efficacy of IV iron products have been conducted [8–12].

Ferumoxytol, a colloidal iron oxide, is an IV iron product approved for the treatment of IDA in adult patients with CKD in the US and Canada as Feraheme® (ferumoxytol) Injection and, at the time of this analysis, was marketed in the US as Feraheme and in the European Union and Switzerland as Rienso® (ferumoxytol) [13]. Ferumoxytol has been investigated for the broad indication of IDA in those who have failed or who are intolerant to oral iron therapy. Unlike most other IV iron products, a full course of ferumoxytol therapy (1.02 g) requires only two IV administrations of 510 mg, delivered between 3 and 8 days apart. Iron sucrose (Venofer®) is approved in the US as an iron replacement product for the treatment of IDA in patients with CKD. Iron sucrose is administered in small doses as a slow IV injection or longer infusion and requires the administration of multiple doses. At the time of this publication, there were little data on the efficacy and safety of iron replacement therapy in patients with various stages of renal function. Thus, the primary objective of this analysis was to provide a deeper understanding of the comparative safety and efficacy of ferumoxytol and iron sucrose across all stages of renal function, from normal kidney function to end-stage CKD.

## Methods

Two recently completed clinical trials compared the efficacy and safety of ferumoxytol with iron sucrose for the treatment of IDA in adults with CKD either on or not on dialysis (**CKD-201**; ClinicalTrials.gov identifier: NCT01052779), and in adults with IDA of any underlying cause and a history of unsatisfactory oral iron therapy or in whom oral iron could not be used (**IDA-302**; ClinicalTrials.gov identifier: NCT01114204). Here, we report the pooled safety and efficacy results of these two randomized, controlled studies with similar study designs to better characterize the safety and efficacy of these IV iron products across all stages of renal function, from normal kidney function to end-stage CKD, as categorized by estimated glomerular filtration rate (eGFR) levels of ≥90, 60 to <90, 30 to <60, 15 to <30, and <15 mL/min.

This post-hoc analysis included pooled data from all patients in the Intent-to-Treat (ITT) populations of two randomized, open-label, controlled clinical trials (**CKD-201** and **IDA-302**) of similar design (*N* = 767). Full details of the study designs and results for the overall patient populations were presented separately in the original two papers [14, 15]. Both studies were conducted in accordance with the ethical principles of Good Clinical Practice and in compliance with the Declaration of Helsinki. The study protocols were reviewed and approved by the institutional review boards or ethics committees at each study site (Additional file 1). Ethical approval for the current study was not required, as it is a post-hoc analysis of pooled data from two previously published clinical trials. All patients provided written informed consent prior to study entry.

The **CKD-201** study included adults aged ≥18 years with CKD on or not on hemodialysis. Patients were required to have a serum hemoglobin (Hgb) <11.0 and ≥7 g/dL and a transferrin saturation (TSAT) <30 % [14]. The **IDA-302** study included patients who had various primary underlying conditions associated with IDA (e.g., abnormal uterine bleeding, cancer, GI disorders, postpartum anemia, and other conditions) with normal (eGFR >90 mL/min), mild (eGFR of 60–90 mL/min), and moderate (eGFR of 30–59 mL/min) decreased renal function, but excluded those with severe (eGFR < 30 mL/min) kidney disease. Patients in **IDA-302** included adults aged ≥18 years with a Baseline Hgb >7 to <10 g/dL and a TSAT <20 % who had either failed oral iron therapy or were intolerant to oral iron [15].

There were a total of 162 patients from the **CKD-201** study and 605 patients from the **IDA-302** study. Overall, 486 patients received ferumoxytol and 281 received iron sucrose. Both studies included a 14-day screening period. Patients in the Ferumoxytol Treatment Group received a 510-mg IV dose at the Baseline visit (Day 1) followed by a second IV dose 2 to 8 days later (Week 1) as a rapid IV injection of 17 mL at a rate not to exceed 1 mL/sec. Patients were observed weekly until the end of the 5-week treatment period (Weeks 2–5). Patients randomized to the Iron Sucrose Treatment Group received a cumulative dose of 1 g as 10 IV doses of 100 mg (hemodialysis patients, **CKD-201**) within 3 weeks or five IV doses of 200 mg on five non-consecutive days over a 14-day period (non-dialysis CKD patients, **CKD-201**; all patients, **IDA-302**). Iron Sucrose was given as an infusion or slow injection. Infusions were given at 25 mg for the first 15 min; if no adverse events (AEs) occurred, the remaining dose was given at a rate not exceeding 100 mg over 15 min. Slow injections were given at 1 mL (20 mg) over 1–2 min; if no AEs occurred, the remaining dose was given. Blood samples were collected at Screening, Baseline, and weekly Visits 2 to 5 to assess efficacy (Hgb, TSAT, other iron measures).

The safety population included all randomized patients who had any exposure to study drug and was based on actual treatment received. The ITT population included any randomized patient who had any exposure to study drug (IV ferumoxytol or IV iron sucrose) and was based upon randomized treatment assignment. The safety and ITT populations were identical in this analysis.

The safety analysis included descriptive summaries of overall AEs, serious AEs (SAEs), study drug-related AEs, AEs resulting in study drug discontinuation, and AEs of Special Interest (AESIs; predefined as moderate-to-severe hypotension occurring on the day of dosing and moderate-to-severe hypersensitivity reactions) and Composite Cardiovascular AEs (predefined as nonfatal myocardial infarction, heart failure, moderate-to-severe hypertension, and hospitalization due to any cardiovascular event). The efficacy analysis focused on mean change in Hgb from Baseline to Week 5, which was the primary efficacy endpoint in both individual studies. The treatment difference in the Hgb change was analyzed using an analysis of covariance model adjusting for Baseline Hgb, primary underlying conditions, and dialysis status.

## Results

### Baseline characteristics and patient disposition

**Fig. 1:**
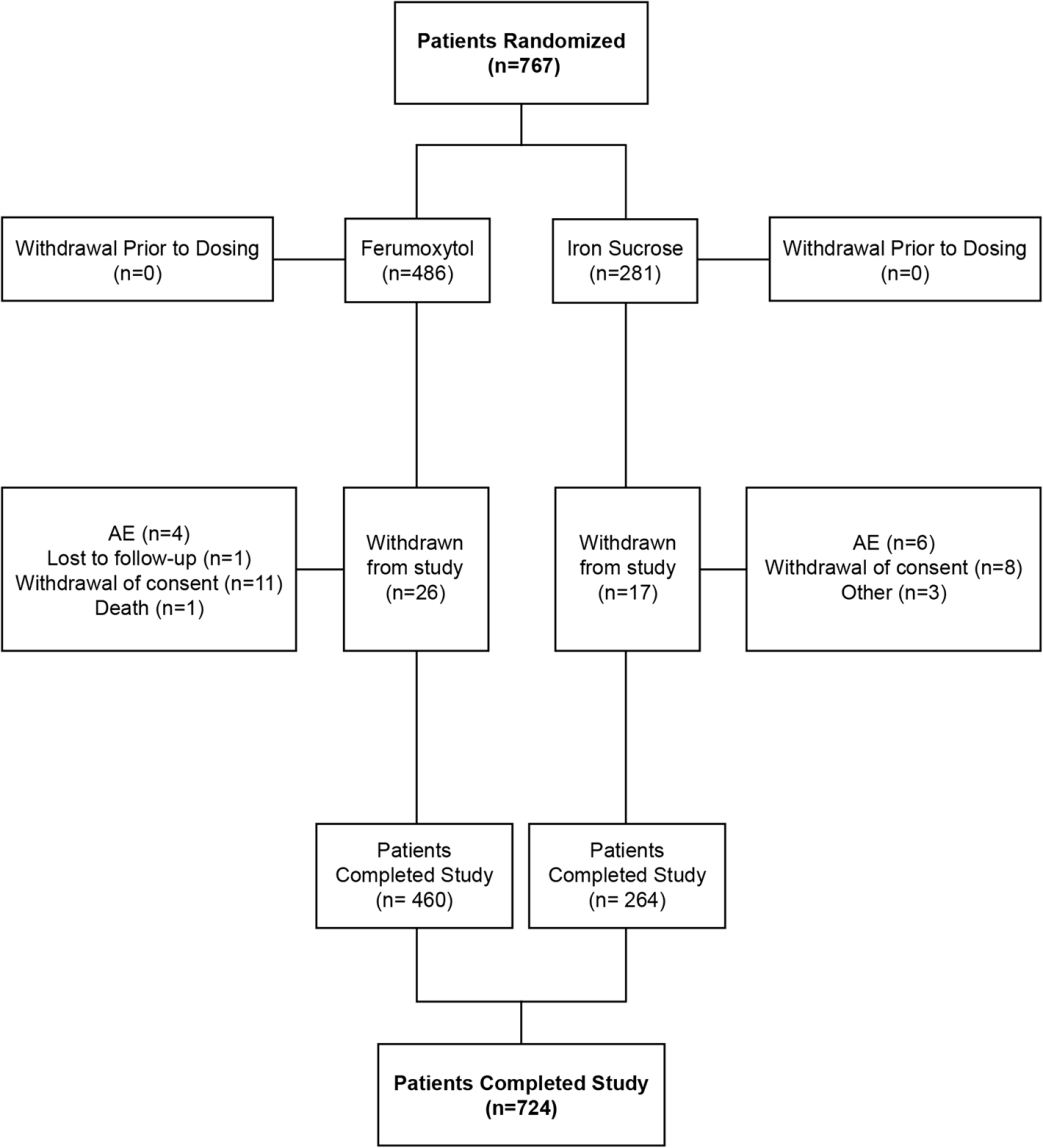
Patient flow diagram. AE, adverse event

The demographic and baseline characteristics for the Ferumoxytol and Iron Sucrose Treatment Groups were comparable (Table 1). The majority of the study population was comprised of women (75.9 %). The mean (± standard deviation [SD]) age was 51.1 ± 15.9 years and most patients were white (81.4 %). The following differences were noted in the characteristics between the two study populations: The mean age of patients in **CKD-201** was 14.4 years greater and the mean weight was 16.3 kg greater. More patients in CKD-201 were receiving treatment with an erythropoiesis-stimulating agent than those from **IDA-302** (52 % of patients in **CKD-201** vs. 1 patient of 605 in **IDA-302**). In addition, a greater percentage of patients from **IDA-302** had more severe anemia on study entry with 33 % having a Baseline Hgb value ≤8.5 g/dL, while only 13 % of patients from **CKD-201** had a Baseline Hgb ≤9 g/dL. Approximately one-half of the study population (52.9 %) had normal renal function (eGFR >90 mL/min), and the distribution of patients classified by eGFR category (i.e., eGFR ≥90, 60 to <90, 30 to <60, 15 to <30, and <15 mL/min) is summarized in Table 1. Mean Baseline values for iron parameters by Baseline CKD stage are summarized in Table 2, with an overall Baseline mean Hgb of 9.1 (±1.04) g/dL in the Ferumoxytol Treatment Group and 9.2 (±1.11) g/dL in the Iron Sucrose treatment group. Patient disposition is illustrated in Fig. 1.

**Table 1:** Demographic and baseline characteristics of pooled treatment groups (total study population - safety population)^a^

	Ferumoxytol Treatment Group (*n* = 486)	Iron Sucrose Treatment Group (*n* = 281)	Total (*N* = 767)
Mean age, years (SD)	50.1 (15.75)	52.8 (16.06)	51.1 (15.90)
Mean weight, kg (SD)	70.9 (16.07)	76.0 (20.29)	72.8 (17.89)
Mean height, cm (SD)	165.8 (8.17)	166.5 (8.18)	166.0 (8.18)
Sex, n (%)			
Female	383 (78.8)	199 (70.8)	582 (75.9)
Male	103 (21.2)	82 (29.2)	185 (24.1)
Race, n (%)			
American Indian/Alaskan Native	0 (0.0)	1 (0.4)	1 (0.1)
Asian	51 (10.5)	18 (6.4)	69 (9.0)
Black/African American	27 (5.6)	16 (5.7)	43 (5.6)
Native Hawaiian/other Pacific Islander	2 (0.4)	1 (0.4)	3 (0.4)
White	390 (80.2)	234 (83.3)	624 (81.4)
Other/multiracial	16 (3.3)	11 (3.9)	27 (3.5)
Ethnicity, n (%)			
Hispanic/Latino	12 (2.5)	13 (4.6)	25 (3.3)
Not Hispanic/Latino	474 (97.5)	268 (95.4)	742 (96.7)
CKD stage, n (%)			
Stage 1 (eGFR ≥90 mL/min)	280 (57.6)	126 (44.8)	406 (52.9)
Stage 2 (eGFR 60 to <90 mL/min)	104 (21.4)	59 (21.0)	163 (21.3)
Stage 3 (eGFR 30 to <60 mL/min)	41 (8.4)	33 (11.7)	74 (9.6)
Stage 4 (eGFR 15 to <30 mL/min)	23 (4.7)	20 (7.1)	43 (5.6)
Stage 5 (eGFR <15 mL/min)	38 (7.8)	42 (14.9)	80 (10.4)
Unknown	0 (0.0)	1 (0.4)	1 (0.1)
Dialysis status, n			
Hemodialysis	34	36	70
ESA use, n (%)	45 (9.3)	40 (14.2)	85 (11.1)

*ESA* erythropoiesis-stimulating agent, *eGFR* estimated glomerular filtration rate, *Hgb* hemoglobin, *SD* standard deviation

^a^Baseline values obtained Day 1 prior to injection of study drug

**Table 2:** Baseline laboratory values by CKD stage, mean (SD)

CKD Stage	Hgb, g/dL		TSAT, %		Ferritin, μg/L	
	Ferumoxytol Treatment Group	Iron Sucrose Treatment Group	Ferumoxytol Treatment Group	Iron Sucrose Treatment Group	Ferumoxytol Treatment Group	Iron Sucrose Treatment Group
All stages	9.1 (1.04) (*n* = 486)	9.2 (1.11) (*n* = 280)	8.7 (12.93) (*n* = 486)	9.4 (12.40) (*n* = 280)	68.8 (170.04) (*n* = 481)	88.6 (184.48) (*n* = 279)
Stage 1 (eGFR ≥90 mL/min)	8.8 (0.95) (*n* = 280)	8.8 (0.98) (*n* = 126)	5.8 (11.25) (*n* = 280)	4.3 (5.21) (*n* = 126)	14.1 (39.76) (*n* = 276)	15.4 (53.11) (*n* = 125)
Stage 2 (eGFR 60 to <90 mL/min)	9.1 (1.0) (*n* = 104)	8.9 (0.89) (*n* = 59)	6.6 (5.7) (*n* = 104)	5.1 (3.47) (*n* = 59)	55.4 (138.26) (*n* = 103)	22.7 (80.21) (*n* = 59)
Stage 3 (eGFR 30 to <60 mL/min)	9.3 (0.86) (*n* = 41)	9.5 (0.88) (*n* = 33)	13.4 (18.31) (*n* = 41)	16.1 (22.18) (*n* = 33)	106.6 (168.37) (*n* = 41)	106.5 (157.15) (*n* = 33)
Stage 4 (eGFR 15 to <30 mL/min)	9.8 (0.84) (*n* = 23)	10.0 (0.76) (*n* = 20)	21.7 (19.41) (*n* = 23)	17.0 (6.49) (*n* = 20)	152.3 (123.03) (*n* = 23)	141.4 (158.16) (*n* = 20)
Stage 5 (eGFR <15 mL/min)	10.2 (1.09) (*n* = 38)	10.2 (1.10) (*n* = 42)	22.3 (12.91) (*n* = 38)	21.7 (14.76) (*n* = 42)	411.3 (348.97) (*n* = 38)	359.7 (287.65) (*n* = 42)

*eGFR* estimated glomerular filtration rate, *Hgb* hemoglobin, *SD* standard deviation, *TSAT* transferrin saturation

Approximately 94 % of patients in each treatment group completed the study. The mean total cumulative doses of IV iron (mg) in the Ferumoxytol and Iron Sucrose Treatment Groups were comparable (1005.0 ± 85.61 and 966.0 ± 142.6 mg, respectively).

### Safety

**Fig. 2:**
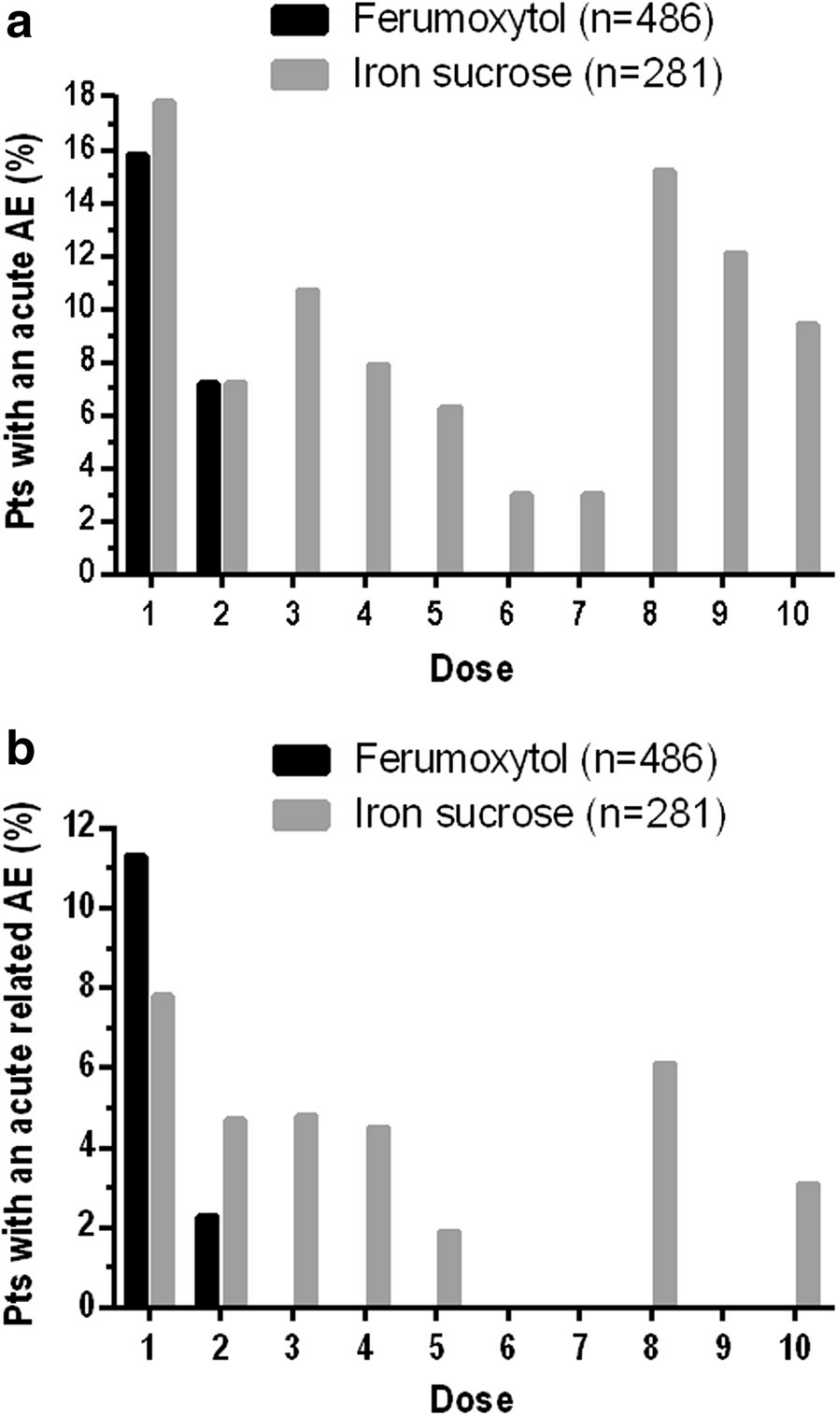
Adverse events (AEs) within 24 h post-dosing. **a** All treatment-emergent AEs by treatment and dose number. **b** All treatment-related AEs by treatment and dose number. Pts, patients

The pooled safety population (i.e., ITT population) included 486 patients in the Ferumoxytol Treatment Group and 281 in the Iron Sucrose Treatment Group. The overall incidence of all AEs was 42.4 % in the Ferumoxytol Treatment Group and 50.2 % in the Iron Sucrose Treatment Group. The incidence of treatment-related AEs was similar (13.6 and 16.0 % for ferumoxytol and iron sucrose, respectively; Table 3). The most frequent AEs were headache (4.5 %) and nausea (3.5 %) in the Ferumoxytol Treatment Group, and dysgeusia (5.0 %) and headache (4.6 %) in the Iron Sucrose Treatment Group (Table 3). Overall AEs and treatment-related AEs occurring within 24 h of each dose are summarized in Fig. 2. Frequencies of AEs were generally similar between the Ferumoxytol and Iron Sucrose Treatment Groups during Weeks 1 and 2, although the Iron Sucrose Treatment Group continued to experience AEs with subsequent administrations.

**Table 3:** Treatment-emergent AEs and incidence of Treatment-emergent AEs occurring in ≥2 % of patients (safety population)

	Ferumoxytol Treatment Group (*n* = 486)	Iron Sucrose Treatment Group (*n* = 281)	Total (*N* = 767)
AE Summary			
All AEs	206 (42.4)	141 (50.2)	347 (45.2)
Treatment-related AEs	66 (13.6)	45 (16.0)	111 (14.5)
SAEs	24 (4.9)	11 (3.9)	35 (4.6)
Treatment-related SAEs	3 (0.6)	1 (0.4)	4 (0.5)
AEs of Special Interest^a^	12 (2.5)	15 (5.3)	27 (3.5)
Cardiovascular AEs^b^	6 (1.2)	3 (1.1)	9 (1.2)
AEs resulting in temporary discontinuation of study drug	3 (0.6)	4 (1.4)	7 (0.9)
AEs resulting in permanent discontinuation of study drug	7 (1.4)	9 (3.2)	16 (2.1)
AEs resulting in study discontinuation	4 (0.8)	6 (2.1)	10 (1.3)
Death	1 (0.2)	0 (0.0)	1 (0.1)
Treatment-emergent AEs occurring in ≥2 % of patients in any treatment group by decreasing incidence in the Ferumoxytol Treatment Group			
Headache	22 (4.5)	13 (4.6)	35 (4.6)
Nausea	17 (3.5)	10 (3.6)	27 (3.5)
Dizziness	10 (2.1)	5 (1.5)	15 (2.0)
Dysgeusia	10 (2.1)	14 (5.0)	24 (3.1)
Peripheral edema	5 (1.0)	8 (2.8)	13 (1.7)
Urinary tract infection	5 (1.0)	9 (3.2)	14 (1.8)
Muscle spasms	5 (1.0)	6 (2.1)	11 (1.4)
Vomiting	4 (0.8)	6 (2.1)	10 (1.3)
Hypotension	3 (0.6)	10 (3.6)	13 (1.7)
Pyrexia	2 (0.4)	7 (2.5)	9 (1.2)

Data are presented as n (%). AE: adverse event; SAE: serious adverse event

^a^Includes hypotension and hypersensitivity

^b^Includes myocardial infarction, heart failure, moderate to severe hypertension, and hospitalization due to any cardiovascular cause

Discontinuation due to AEs occurred at comparable rates in the Ferumoxytol (0.8 %) and Iron Sucrose (2.1 %) Treatment Groups. Similarly, the proportion of patients experiencing AESIs (i.e., hypotension and hypersensitivity) was comparable between treatment groups (ferumoxytol, 2.5 %; iron sucrose, 5.3 %; Table 3). Five of the AESIs in the Iron Sucrose Treatment Group occurred at Weeks 3 through 10 (i.e., after all ferumoxytol administrations had been completed). One patient death occurred in the Ferumoxytol Treatment Group. The patient had a pancreatic tumor causing duodenal obstruction and died postoperatively; this was considered unrelated by the Investigator. Composite Cardiovascular AEs occurred at a similar incidence in both treatment groups (ferumoxytol, 1.2 %; iron sucrose, 1.1 %). Similarly, rates of SAEs and treatment-related SAEs were comparable between treatment groups (ferumoxytol, 4.9 and 0.6 %; iron sucrose 3.9 and 0.4 %, respectively). Four patients (0.5 %) experienced an SAE within 24 h of the first dose of IV iron (ferumoxytol, *n* = 1 [0.2 %]; iron sucrose, *n* = 3 [1.1 %]). SAEs occurring within 24 h of the first dose of IV iron were considered treatment-related in one patient in each group (ferumoxytol, 0.2 %-moderate intensity anaphylactic-type reaction that resolved on the day of dosing; iron sucrose, 0.4 %-moderate intensity hypotension treated with volume and Trendelenburg positioning).

### Efficacy

**Fig. 3:**
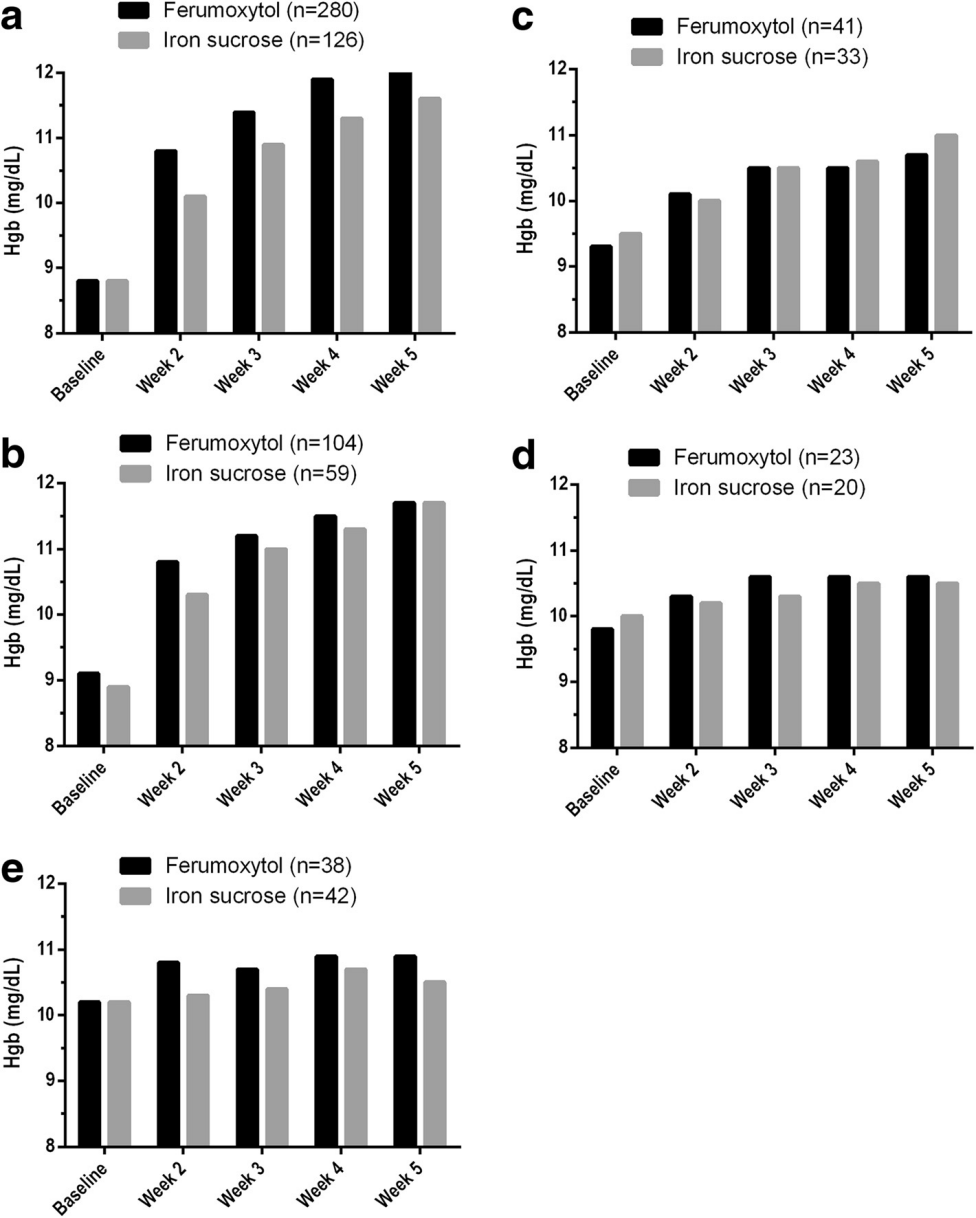
Mean hemoglobin (Hgb) levels across renal functions. **a** eGFR ≥90 mL/min; (**b**) eGFR 60 to <90 mL/min; (**c**) eGFR 30 to <60 mL/min; (**d**) eGFR 15 to <30 mL/min; and (**e**) eGFR <15 mL/min at Weeks 2, 3, 4, and 5 in patients receiving ferumoxytol or iron sucrose therapy (Intent-to-Treat population). eGFR, estimated glomerular filtration rate

Hgb levels increased from Baseline until the end of the study in both treatment groups; Fig. 3 summarizes mean Hgb levels over time by CKD stage. For patients with an eGFR ≥90 mL/min (normal renal function), mean (± SD) Hgb increased from 8.8 (±0.95) mg/dL at Baseline to 12.1 (±1.3) mg/dL at Week 5 (a change of 3.3 g/dL) in the Ferumoxytol Treatment Group, while mean Hgb increased from 8.8 (±0.98) mg/dL to 11.6 (±1.33) mg/dL (a change of 2.8 g/dL) for those in the Iron Sucrose Treatment Group. For patients with normal renal function, ferumoxytol treatment resulted in significantly greater increases in mean Hgb levels compared with iron sucrose treatment at Weeks 2, 3, 4, and 5 (*p* < 0.001 vs. iron sucrose for all; Fig. 3a). Least Squares (LS) mean treatment differences between the Ferumoxytol and Iron Sucrose Treatment Groups for Hgb levels ranged from 0.50 to 0.76 g/dL. Mean Hgb values over time for the other CKD categories are illustrated in Fig. 3b–e. Ferumoxytol-treated patients with eGFRs of 60 to <90 mL/min and <15 mL/min at Week 2 also had a significantly greater increase in Hgb levels compared with iron sucrose-treated patients (*p* = 0.009 and *p* = 0.012, respectively, vs. iron sucrose); there were no significant treatment differences in Hgb levels between treatment groups in the other categories of renal function (Fig. 3b).

Mean Baseline TSAT increased with declining renal function (Table 2). Treatment with both ferumoxytol and iron sucrose resulted in increased mean TSAT values over the 5-week study period (Table 4). LS mean increases in TSAT from Baseline to Week 5 were significantly greater for those receiving ferumoxytol compared with those receiving iron sucrose for the eGFR category of 60 to <90 mL/min (15.9 vs. 11.3 %; *p* = 0.001). There were no significant differences between Ferumoxytol and Iron Sucrose Treatment Groups at Week 5 for any of the other CKD categories.

**Table 4:** Mean change in TSAT and serum ferritin over time by CKD stage

CKD Stage	TSAT, % (SD)		Ferritin, μg/L (SD)	
	Ferumoxytol Treatment Group	Iron Sucrose Treatment Group	Ferumoxytol Treatment Group	Iron Sucrose Treatment Group
Stage 1 (eGFR ≥90 mL/min)	(*n* = 280)	(*n* = 126)	(*n* = 280)	(*n* = 126)
Baseline	5.8 (11.25)	4.3 (5.21)	14.1 (39.76)	15.4 (53.11)
Week 2	26.1 (19.59)	15.8 (11.90)	291.1 (192.69)	172.5 (245.93)
Week 3	24.1 (11.54)	19.8 (15.38)	179.7 (157.02)	157.3 (211.08)
Week 4	22.7 (10.95)	19.2 (13.81)	123.7 (124.20)	124.0 (211.93)
Week 5	22.9 (21.75)	19.1 (14.77)	98.3 (118.96)	96.4 (185.27)
Stage 2 (eGFR 60 to <90 mL/min)	(*n* = 104)	(*n* = 59)	(*n* = 104)	(*n* = 59)
Baseline	6.6 (5.7)	5.1 (3.47)	55.4 (138.26)	22.7 (80.21)
Week 2	26.2 (11.41)	25.8 (51.56)	442.0 (398.63)	215.0 (226.93)
Week 3	24.3 (10.39)	17.2 (7.81)	325.6 (385.93)	196.6 (212.70)
Week 4	23.9 (11.17)	18.9 (12.46)	277.9 (394.82)	166.2 (254.32)
Week 5	22.5 (10.35)	16.5 (6.66)	247.2 (471.24)	127.5 (214.99)
Stage 3 (eGFR 30 to <60 mL/min)	(*n* = 41)	(*n* = 33)	(*n* = 41)	(*n* = 33)
Baseline	13.4 (18.31)	16.1 (22.18)	106.6 (168.37)	106.5 (157.15)
Week 2	26.6 (13.27)	21.8 (22.81)	629.3 (507.21)	338.6 (296.76)
Week 3	25.0 (11.45)	18.9 (8.16)	518.1 (425.74)	373.0 (321.68)
Week 4	23.2 (11.01)	19.2 (7.78)	452.5 (443.14)	314.8 (284.35)
Week 5	23.1 (12.15)	19.4 (7.86)	426.0 (433.68)	312.0 (284.41)
Stage 4 (eGFR 15 to <30 mL/min)	(*n* = 23)	(*n* = 20)	(*n* = 23)	(*n* = 20)
Baseline	21.7 (19.41)	17.0 (6.49)	152.3 (123.03)	141.4 (158.16)
Week 2	30.6 (9.71)	24.3 (7.14)	720.7 (280.38)	520.2 (244.56)
Week 3	28.6 (13.22)	24.0 (5.85)	632.8 (324.78)	529.6 (244.92)
Week 4	32.2 (15.81)	25.7 (7.18)	552.4 (327.53)	440.5 (228.45)
Week 5	26.3 (6.34)	23.1 (5.79)	514.6 (285.12)	407.0 (231.30)
Stage 5 (eGFR <15 mL/min)	(*n* = 38)	(*n* = 42)	(*n* = 38)	(*n* = 42)
Baseline	22.3 (12.91)	21.7 (14.76)	411.3 (348.97)	359.7 (287.65)
Week 2	34.6 (16.18)	25.4 (12.87)	915.8 (372.92)	603.4 (338.87)
Week 3	32.2 (12.81)	24.7 (7.44)	959.0 (605.92)	675.2 (339.54)
Week 4	30.4 (12.57)	27.7 (13.27)	831.7 (452.94)	682.2 (391.21)
Week 5	28.7 (8.82)	27.9 (15.80)	788.4 (427.21)	618.9 (355.47)

*CKD* chronic kidney disease, *GFR* glomerular filtration rate, *SD* standard deviation, *TSAT* transferrin saturation

Low serum ferritin is a marker for IDA, but an elevated serum ferritin can be a marker of either increased iron levels or inflammation, the latter of which can mask the presence of IDA [7]. Overall, mean Baseline values for ferritin levels in the Ferumoxytol and Iron Sucrose Treatment Groups were similar with increasing Baseline ferritin levels with declining renal function (Table 2). As for other iron parameters, treatment with either ferumoxytol or iron sucrose was associated with increased ferritin levels over time in all eGFR categories (Table 4).

## Discussion

The current analysis pooled data from two randomized, controlled clinical studies of IV iron treatment in adults with IDA, which provided the opportunity to more thoroughly analyze a larger number of patients across the full range of Baseline renal function. It also allowed for a comparative analysis of the Hgb response to IV iron treatment based on underlying renal function. Across all stages of renal function—from normal to end-stage CKD—the comparison of the safety and efficacy of ferumoxytol versus iron sucrose treatment demonstrated consistent findings. Overall, the incidences of all AEs were numerically lower in the Ferumoxytol Treatment Group compared to those treated with iron sucrose. This may be related to the risk of AEs associated with each instance of an IV administration, as suggested by the continued occurrence of AEs (including AESIs) after each dose in patients in the Iron Sucrose Treatment Group who received up to 10 individual doses. The frequencies of drug-related AEs and AE-related treatment discontinuations were slightly lower in the Ferumoxytol Treatment Group compared with the Iron Sucrose Treatment Group, while SAEs and treatment-related SAEs were slightly higher with ferumoxytol than iron sucrose. It is important to emphasize that, given the low overall rates, no statistical comparisons were appropriate. Overall, both agents had a comparable rate of SAEs, treatment-related SAEs, and cardiovascular AEs, and no new safety signals were identified. These results are consistent with those observed in the original Phase 3 studies [14, 15].

Hgb levels were increased in both the Ferumoxytol and Iron Sucrose Treatment Groups at the end of the study. Patients in all of the CKD categories were shown to benefit from iron therapy, although those with the most severe renal dysfunction achieved somewhat less of an Hgb response. Compared with iron sucrose, ferumoxytol treatment resulted in a significantly greater increase in Hgb at all time points assessed in those with an eGFR ≥90 mL/min. Statistically significant differences between treatment groups were not observed for patients with decreased levels of renal function for the majority of time points.

A low serum ferritin is indicative of iron deficiency, and elevated levels may be indicative of increased iron stores or iron overload. However, since ferritin is also an acute phase reactant, it is also a surrogate marker for inflammation and may be elevated due to chronic inflammation even in the presence of IDA [7]. Thus, while ferritin levels can be used to assess iron levels and the efficacy of active therapies, they must be used with caution in patients with CKD, especially with those on dialysis [7]. In this study, Baseline levels were increased in patients with reduced renal function. Treatment with either ferumoxytol or iron sucrose was shown to produce increases in serum ferritin across all CKD categories. The increases in TSAT levels were significantly greater with ferumoxytol than with iron sucrose in all CKD categories as early as Week 2 and/or 3 following the start of treatment, indicating that ferumoxytol is associated with a faster replenishment of iron stores available for erythropoiesis.

A limitation of this study is the retrospective nature of the analysis. Another limitation of this pooled analysis is that data were pooled across different populations. In particular, patients in **CKD-201** were older and heavier compared with those from study **IDA-302**. Patients in **CKD-201** were also less likely to have more severe anemia than those in **IDA-302**, most likely because CKD patients routinely receive aggressive therapy for anemia (e.g., iron therapy, erythropoiesis-stimulating agents).

## Conclusions

This pooled analysis of two randomized, controlled clinical trials of IV iron treatment provides the opportunity to compare the safety and efficacy of two IV iron replacement products in a large population of patients with IDA and varying degrees of renal function. Overall, the efficacy of ferumoxytol was shown to be comparable, and at times greater, to that of iron sucrose for increasing Hgb across the spectrum of renal function, from normal to patients with CKD on dialysis. Overall, mean Hgb increased in both treatment groups, regardless of degree of renal insufficiency. Although patients with worse renal function did not respond as well to IV iron therapy with either drug compared to those with normal renal function, patients in all CKD categories experienced improved iron parameters. In this pooled analysis, both ferumoxytol and iron sucrose were shown to have comparable safety profiles consistent with those observed in the original studies.

## Abbreviations

AE, adverse event; AESI, adverse event of special interest; CKD, chronic kidney disease; eGFR, estimated glomerular filtration rate; ESA, erythropoiesis-stimulating agent; GI, gastrointestinal; Hgb, hemoglobin; IDA, iron deficiency anemia; ITT, intent to treat; IV, intravenous; LS, least squares; SAE, serious adverse event; SD, standard deviation; TSAT, transferrin saturation

## Additional file

Additional file 1: List of institutional review boards (IRBs). IRBs that approved the CKD-201 and IDA-302 studies. (DOCX 39 kb)
