# Supplementary material for: Ferumoxytol versus iron sucrose treatment: a post-hoc analysis of randomized controlled trials in patients with varying renal function and iron deficiency anemia
Source: BMC Hematol. 2016 Jul 26;16:20. doi: 10.1186/s12878-016-0060-x (PMC4960805; doi:10.1186/s12878-016-0060-x)
Supplement: Additional file 1: — List of institutional review boards (IRBs). IRBs that approved the CKD-201 and IDA-302 studies. (DOCX 39 kb) [file 12878_2016_60_MOESM1_ESM.docx]

*The following IRBs approved the CKD-201 study and the IDA-302 study.*

| CKD-201 Study | Quorum IRB was the central IRB that approved this study |
| --- | --- |
| IDA-302 Study | IDA-302 study was approved by the following local IRBs:   - Melbourne Health Human Research Ethics Committee - Royal Adelaide Hospital Research Ethics Committee - Sydney West Area Health Service Human Research Ethics Committee - Centre for Digestive Disease Human Research Ethics Committee - Belberry Human Research Ethics Committee - Seoul National University Hospital Institutional Review Board - Gangnam Severance Hospital Institutional Review Board - Gachan University Gil Hospital Institutional Review Board - Institutional Review Board of Asan Medical Center - Keimyung University Dongsan Hospital Institutional Review Board - Ajou University Hospital Institutional Review Board - Institutional Review Board of Ewha Womans University Mokdong Hospital - Samsung Medical Center Institutional Review Board - The Catholic University of Korea, St. Vincent’s Hospital Institutional Review Board - Institutional Review Board of Kangdong Sacred Heart Hospital - Inha University Hospital Institutional Review Board - Soonchunhyang University Cheonan Hospital Institutional Review Board |
